# Hypoxia-inducible factor-1α polymorphisms and TSC1/2 mutations are complementary in head and neck cancers

**DOI:** 10.1186/1476-4598-5-3

**Published:** 2006-01-16

**Authors:** Carla Hebert, Kathleen Norris, Pallavi Parashar, Robert A Ord, Nikolaos G Nikitakis, John J Sauk

**Affiliations:** 1 Department of Diagnostic Sciences and Pathology, University of Maryland Baltimore, Baltimore, Maryland 21201-1586, USA; 2 Greenebaum Cancer Center, University of Maryland Baltimore, Baltimore, Maryland 21201-1586, USA; 3 Department of Oral and Maxillofacial Surgery, University of Maryland Baltimore, Baltimore, Maryland 21201-1586, USA

## Abstract

**Background:**

Polymorphisms or mutations in hypoxia inducible factor-1 alpha (HIF-1alpha) that increases its activity and stability under normoxia have recently been identified. Likewise, disruption of the TSC1/TSC2 complex through loss of TSC1 or TSC2 has been shown to result in abnormal accumulation of HIF-1α. Here, we investigate the novel polymorphisms in exon 12, that approximate the oxygen-dependent degradation domain of HIF-1alpha in five cell lines and 28 patients with oral squamous carcinomas. Moreover, we assess for the presence of polymorphisms and mutations in TSC1 and TSC2, to ascertain if dysregulation of such might complement HIF-1alpha expression.

**Results:**

Denaturing high pressure liquid chromatography (DHPLC) analysis on PCR fragments in exon 12 of HIF-1alpha from 28 patients with OSCC revealed that 6 of 28 patients had mismatched heteroduplex patterns. Genomic DNA was extracted from peripheral blood leukocytes and direct sequencing showed that in 5 of the six cases these changes represented polymorphisms while, one case was a somatic mutation. Analyses of TSC1 and TSC2 revealed heteroduplexes in exons: TSC1 exon 17; TSC2 exons 36,40, and 41. The relative levels of HIF-1alpha were significantly greater for tumors possessing a HIF-1alpha polymorphism or mutation within exon 12, whereas tumors possessing a deletion or polymorphism in TSC1/TSC2 displayed a trend for higher levels of HIF-1alpha. Western blot analyses for HIF-1alpha, TSC1 and TSC2 in five SCC cell lines revealed high levels of HIF-1alpha in SCC cells possessing TSC1 and/or TSC2 mutations. Wild-type TSC2 cells targeted with siRNA to TSC2 exhibited increased levels of HIF-1alpha. Transfection of a HIF-1alpha mutant produced higher levels of HIF-1alpha in TSC1/TSC2 mutant cell lines than in wild type cells. TSC1/TSC2 mutant cell lines administered Rapamycin blocked S6 phorphorylation and diminished the levels of HIF-1alpha to those observed in cell lines with wild type TSC1/TSC2.

**Conclusion:**

Dysregulation of the TSC1/TSC2 complex by mutation compliments HIF-1α polymorphisms in the expression of HIF-1alpha in SCC of the head and neck, and may provide biomarkers to predict responses to specific therapies and overall disease prognosis.

## Background

Hypoxia, a frequent effect of solid tumor growth in head and neck cancer and other cancers, serves to generate a cascade of molecular pathways which include angiogenesis, glycolysis, and various cell-cycle control proteins. These cell-salvaging mechanisms can be carried out rapidly by a transcription factor that reacts to hypoxic conditions, the hypoxia-inducible factor-1 (HIF-1) [1]. HIF-1 is a heterodimer consisting of an α subunit and a β subunit, both of which are members of the basic helix-loop-helix Per/Arnt/Sim (PAS) family [2]. Stability of this dimer is dependent to a large extent on oxygen [3,4]. Thus, in the presence of oxygen HIFα family members are hydroxylated on one of two conserved prolyl residues. This is achieved by members of the egg-laying-defective nine (EGLN) family or prolyl hydroxylases (PHD1, PHD2, and PHD3) that achieve hydroxylation, using Fe^2+ ^and ascorbate as cofactors [5,6]. In so doing, prolyl hydroxylation creates a binding site for a ubiquitin ligase complex containing the von Hippel-Lindau (VHL) tumor suppressor protein, which results in HIFα destruction [7-10]. Most recently, OS-9, a ubiquitous cellular protein was shown to be a common partner for HIF-α and the PHDs, as well as to enhance prolyl hydroxylation and degradation of HIF-1α [11]. Conversely, in cells where OS-9 mRNA was targeted for degradation, increased HIF-1α levels and accordingly increased HIF-mediated transcription were observed [11].

HIFα transcriptional activation function is also modulated further by asparagine 803 hydroxylation by the asparagine hydroxylase, factor-inhibiting HIF (FIH), which affects recruitment of the coactivators p300/CBP [12-17]. Interestingly, VHL itself has also been implicated in the direct regulation of HIF-1α transcriptional activity, either by recruiting histone deacetylases or by recruiting other transcriptional repressors such as pVHL-associated KRAB-A domain-containing protein (VHLaK) [18]. Thus, in VHL disease, loss of VHL function results in HIF-1α stabilization and increased expression of HIF-1α target genes as noted above. However, more recently it has become apparent that HIF-1α may be expressed and transcriptionally active during normoxia and high levels of HIF-1α have been observed in some normal tissues and in many tumors in the absence of apparent hypoxia or loss of VHL function [19-21]. In fact, increased levels of HIF-1α have been reported in colon, breast, stomach, pancreas, prostate, kidney, esophagus and head and neck cancers [22-24]. Accordingly, there is accumulating evidence that hypoxic-independent mechanisms are attributable for HIF-1α expression.

Despite the observed importance of HIF-1α stabilization by way of VHL loss of function in human malignancies [25], polymorphisms or mutations in HIF-1α that increase its activity under normoxia have recently been identified. These HIF-1α variants exhibit significantly higher transcription activity than wild-type (WT) HIF-1α, under normoxic conditions (P < 0.02). Furthermore, tumors from HNSCC patients with heterozygous alleles were shown to have significantly increased numbers of microvessels. Thus, the elevated transactivation capacity of variant forms of HIF-1alpha has implied a role of HIF-1α polymorphisms in generating individually different tumor progression profiles [26].

TSC1 and TSC2 constitute the tuberous sclerosis complex and hamartin and tuberin constitute their respective protein products. Hamartin and tuberin have been considered to act as tumor suppressors [27]. The proteins interact with each other and non-truncating mutations that disrupt this complex have been shown to cause tuberous sclerosis [28]. Functionally, hamartin stabilizes tuberin by preventing its ubiquitination. This TSC1/TSC2 complex regulates activity of p70S6 kinase by inhibiting mTOR by way of the PI3K/Akt/mTOR pathway [29]. In so doing TSC1/TSC2 exert translational control of protein synthesis and cell growth. Hamartin and tuberin deficient cells also manifest increased proliferation and reduced expression of the cyclin dependent kinase (CDK) inhibitor p27 [30]. Furthermore, TSC1/TSC2 participate more directly in cell adhesion [31]. Hamartin interacts with the ezrin-radixin-moesin family of cytoskeletal proteins and activates the small GTPase Rho that directs cell adhesion by controlling activation of focal adhesion complexes; at the same time, tuberin appears to mediate E-cadherin directed cell adhesion through the β-catenin signaling pathway [30,32,33].

The suppressor roles of hamaratin and tuberin have been extensively investigated in organs that are affected by the tuberous sclerosis phenotype. More recently, it has been shown that aberrant expression of TSC1 and TSC2 may be present in breast cancers, renal carcinoma, transitional cell carcinoma, basal cell carcinoma, squamous cell carcinoma and shagreen patches and may serve as a prognostic marker in breast cancer [34-37].

Here, we investigate the novel polymorphisms in exon 12, that approximate the oxygen-dependent degradation domain of HIF-1α in 28 patients with oral squamous carcinomas. Moreover, we explore the presence of polymorphisms and mutations in TSC1 and TSC2, downstream suppressors of Akt signaling, to ascertain if dysregulation of such elements might complement HIF-1α expression.

## Results

### Immunocytochemical staining of HI-1α in oral squamous cell carcinomas (OSCCs)

A total of 28 OSCCs were grouped as 8 HIF-1α negative tumors;20 HIF-1α positive tumors. 10 of the 20 positive tumors (50 %) were classified as showing high HIF-1α expression. The frequency of high HIF-1α expression was not apparent with tumor stage or grade according to TNM system (Table I).

**Table 1:** Summary of SCC Tumors

Patient #	Stage	Grade	HIF-1α Score	HIF-1α P/M	TSC1/2 P/M	vHL P/M
1	2	WD	2	NC	Exon 41(TSC2), P/G5346C	NC
2	3	UD	1	NC	NC	NC
3	2	WD	3	NC	NC	NC
4	1	WD	2	NC	Exon 41(TSC2), P/G5346C	NC
5	2	MD	3	Exon 12, P/C1772T	Exon 41(TSC2), P/G5346C	NC
6	4	UD	2	NC	NC	NC
7	1	WD	1	NC	Exon 41(TSC2), P/G5346C	NC
8	2	WD	4	NC	NC	NC
9	1	WD	1	NC	Exon 41(TSC2), P/G5346C	NC
10	2	MD	3	NC	NC	NC
11	3	WD	3	Exon 12, P/C1772T	Exon 41(TSC2), P/G5346C	NC
12	2	MD	1	NC	Exon 41(TSC2), P/G5346C	NC
13	1	WD	1	NC	NC	NC
14	1	WD		NC	NC	NC
15	3	MD	3	Exon 12, P/G1790A	NC	NC
16	2	MD	1	NC	NC	NC
17	2	WD	2	NC	NC	NC
18	2	WD	3	NC	Exon 40 (TSC2) 528_5255 del	NC
19	1	WD	2	NC	NC	NC
20	4	MD	2	NC	NC	NC
21	3	WD	3	Exon 12, P/G1790A	NC	NC
22	2	WD	1	NC	NC	NC
23	2	WD	1	NC	NC	NC
24	3	WD	2	NC	NC	NC
25	4	MD	2	NC	NC	NC
26	2	MD	3	Exon 12, P/C1772T	NC	NC
27	2	WD	2		NC	NC
28	3	WD	4	Exon 12, M/C1771A	Exon 36 (TSC2) 4681 delAG 40 (TSC2) 5238_5255 del 41(TSC2) P/G5346C	NC

WD-Well differentiated;MD-Moderately Well-differentiated; UD-Undifferentiated;NC-No change wild-type; P-polymorphism; M-Mutation; del-deletion

### Polymorphisms and/or mutations in: exon 12 of HIF-1α, exons 1,2, and 3 in vHL and TSC1/TSC2

**Figure 1:**
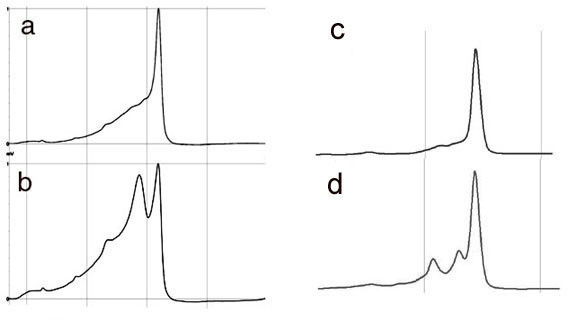
**Representative DHPLC profiles of heteroduplexes. **HIF-1α heteroduplex, left panel and TSC2 heteroduplex, right panel. **a) **Wild-type HIF-1α exon 12, **b) **HIF-1α heteroduplex in exon 12; **c**) wild-type TSC2 exon 40 and **d**) hereroduplex in exon 40 TSC2.

DHPLC analysis on PCR fragments in exon 12 of HIF-1α from 28 patients with OSCC revealed that 6 of 28 patients had mismatched heteroduplex patterns, indicating the existence of polymporphisms or mutations (Figure 1). To identify and confirm the existence of polymorphisms or somatic mutations, sequencing of the PCR fragments from all of the patients and cell lines was performed. These investigations established a base change of C to T at 1772, and G to A change at 1790. The consequences of such were substitution of proline for serine at codon 582 in 4 subjects, and an alanine for threonine at codon 588 in 2 subjects. To determine whether these were somatic mutations or polymorphisms, genomic DNA was extracted from peripheral blood leukocytes. Direct sequencing showed revealed that in 5 of the six cases these changes represented polymorphisms and in one case the G to A change represented a somatic mutation (Table I). DHPLC analysis on PCR fragments in exons 1,2 and 3 of VHL from 28 patients revealed no heteroduplexes. Conversely, the analyses of TSC1 and TSC2 reveal heteroduplexes in the following exons: TSC1 exon 17; TSC2 exons 36,40, and 41. A summary of results is shown in table I.

**Figure 2:**
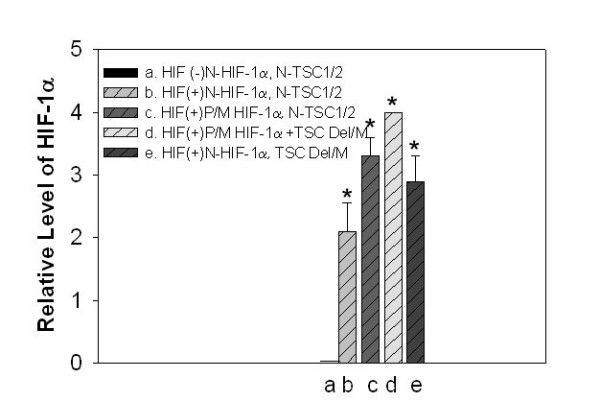
**The relative scores (0–4+) of HIF-1α levels in 28 squamous carcinomas of the tongue analyzes by immunohistochemistry and scanned using NIH image software. a**) HIF-1α negative, **N**, no polyporphisms or mutations identified for HIF-1α, or TSC1 or TSC2; **b**) HIF-1α positive (2.1 ± .45), **N, **no polyporphisms or mutations identified for HIF-1α, or TSC1 or TSC2; **c**) HIF-1α positive (3.3 ± .3), **P/M, **HIF-1α polymorphism or mutation, **N**, no polymorphism or mutation in TSC1 or TSC2; **d**) HIF-1α positive (4.0), **P/M, **HIF-1α polymorphism mutation + TSC deletion or mutation;**e**) HIF-1α positive (2.9 ± .4), **N, **no polyporphisms or mutations identified for HIF-1α, **TSC **deletion or mutation. (*, P < 0.05)

**Figure 3:**
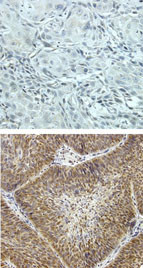
**Immmunohistochemical staining of SCC tumors. **Immunostaining for HIF-1α (SC10790), (Santa Cruz Biotech., Inc. Santa Cruz, CA, USA) was performed on 5 μM sections. Staining was analyzed with horseradish peroxidase-linked goat antimouse (Santa Cruz Biotechnology, Santa Cruz, CA) Tumors lacking HIF-1α (top panel) and tumors that manifest an exon 12 HIF-1α polymorphism and TSC deletions in exon 36 and 40 (bottom panel).

Noteworthy, was that the relative levels of HIF-1α were significantly greater for tumors possessing a HIF-1α polymorphism or mutation within exon 12. Interestingly, tumors possessing a deletion or polymorphism in TSC1/TSC2 displayed a trend for higher levels of HIF-1α and a single tumor with both a HIF-1α polymorphism and an exon 36 and 40 TSC-2 deletion manifest the highest levels of HIF-1α (Figure 2 & 3)

### Western blot analyses for HIF-1α, OS-9, harmartin and tuberin in SCC cell lines

**Figure 4:**
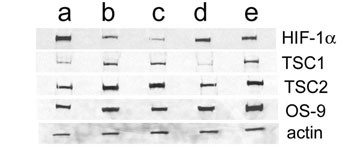
**Western blot for HIF-1α, TSC1, TSC2, OS-9 and Actin. **Cells were grown under normoxic conditions, harvested and analyzed using SDS-PAGE followed by Western blotting as described in *Methods*. The representative lanes are as follows: **a**) SCC-4a, **b**)SCC-9, **c**)SCC-15, **d**) SCC-25b, and **e**)UMBSCC-11.

Western blot analysis for HIF-1α revealed high levels in SCC-4a and SCC-25b while SCC 9 and SCC15 possessed little or no levels of protein at normoxic conditions. However, when the cell lines were grown at in a hypoxic environment of <0.5% oxygen the level of HIF-1α protein were dramatically increased, especially in SCC-9 and SCC-15 cell lines (not shown). The levels of vHL were near equivalent for all of the cell lines (not shown) while the levels of hamartin (TSC 1) were slightly diminish in SCC-4a, and -25b cell lines compared with SCC-9, -15 and SCC-11 cells. Tuberin (TSC2) levels were comparatively diminished in the SCC-4a and SCC-25b cell lines (Figure 4).

**Figure 5:**
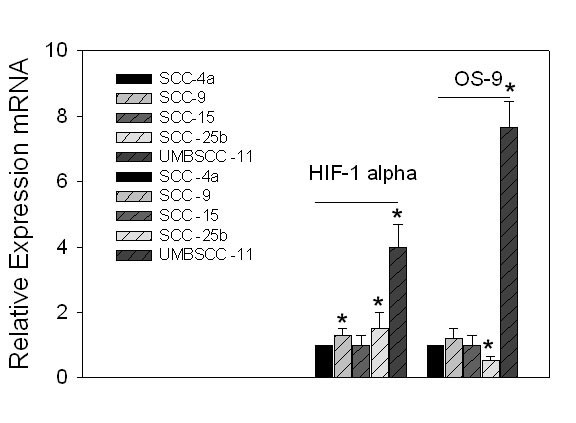
**Real-time PCR analysis for the relative expression of HIF-1α and OS-9 among five SCC cell lines. **Cells were plated in 6-well plates using a density of 5 × 10^4 ^cells/well and were allowed to grow to 80% confluence. The cells were washed twice with cold PBS, lysed in RIPA buffer for 10 min and scraped. The extracts were centrifuged and equivalent amounts of protein (50 μg) were then separated by SDS-PAGE and then transferred to polyvinylidene difluoride membranes. The membranes were blocked for 1 h in blocking buffer, which was subsequently replaced by the primary antibody in blocking buffer, overnight at 4°C. After incubation, the membranes were washed three times in washing buffer. Primary antibody was detected using horseradish peroxidase-linked goat antimouse (Santa Cruz Biotechnology, Santa Cruz, CA) or goat antirabbit IgG antibody (Santa Cruz Biotechnology) and visualized with SuperSignal West Pico chemiluminescent substrate (Pierce, Rockford, IL). The bands were scanned and quantified using NIH image software. The levels of HIF-1α were: SCC- 4a (1.0 ± 0), SCC- 9 (1.3 ± .2); SCC-15 (1.0 ± .3), SCC-25b (1.5 ± .5), and UMBSCC-11 (4.0 ± .7). The levels of OS-9 expression were:SCC-4a (1.0 ± 0), SCC- 9 (1.2 ± .3); SCC-15 (1.0 ± .3), SCC-25b (.5 ± .1), and UMBSCC-11 (7.6 ± .8). (*, P < 0.05)

Recognizing that the OS-9 enhances prolyl hydroxylation and degradation of HIF-α, while in cells where OS-9 is knocked-down, increased HIF-1α levels and increased HIF-mediated transcription occur [11], we determined the level of expression of OS-9 among the five cell lines. These studies surprisingly revealed that the levels of HIF-1α did not correlate well with the level of OS-9 expression and that in the UMSCC-11 cell line the levels of OS-9 were the greatest despite maintaining levels of HIF-1α during normoxia (Figure 4 & 5).

### TSC2 mutants and TSC2 targeted cells exhibit increased levels of HIF-1α

**Figure 6:**
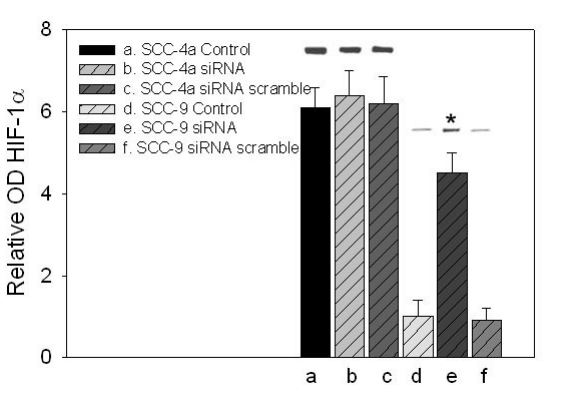
**The relative optical density of HIF-1α proteins from Western blots**. SCC-4a and SCC-9 cells were treated with 20 ng of siRNA directed against TSC2. A TSC2 scramble was included as an internal control and verified by Western blotting (not shown). The cells were exposed for 30 minutes to EGF and then lysed. Representative gel bands corresponding to HIF-1α are immediately depicted above the bar graph. (*, P < 0.05)

**Figure 7:**
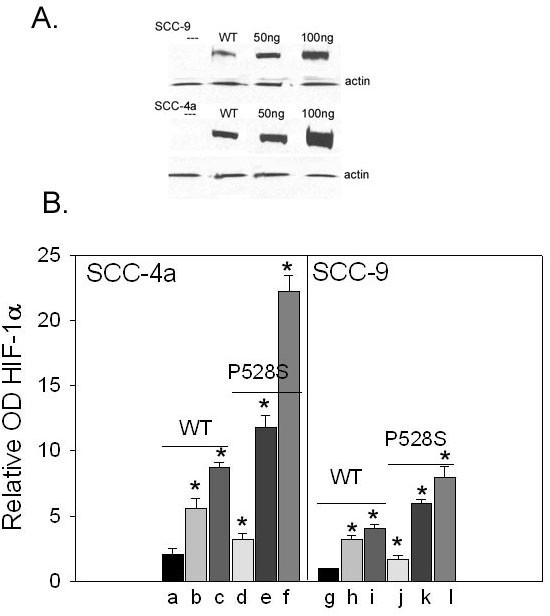
a, b : **Mutant HIF-1α P582S has enhanced protein levels in SCC cells possessing TSC1/TSC2 mutations. **Full length, HA-tagged wild-type HIF-1α and P582S mutant in pcDNA3 plasmids were transfected into SCC-4a and SCC-9 cells. **a**: Immunoblot of HIF-1α with anti-HAepitope-tag antibody. Anti-actin antibody was used as control for normalization of protein samples. **b**:Relative optical density of Western blots bands of HIF-1α wild-type and P582S mutant in SCC-1a and SCC-9 cells. 10 ng, 50 ng, and 100 ng plasmid: a, b, c and g, h, i (WT), 10 ng, 50 ng, and 100 ng plasmid: d, e, f and j, k, l (P582S mutant). (*, P < 0.05)

Although SCC4a and SCC 25b cells manifest high levels of HIF-1α protein expression in normoxia, analysis of the gene did not reveal any polymorphisms or mutation in these or any of the other cell lines. Conversely, SCC-4a revealed a frameshift deletion in exon 17 of TSC1and both SCC4 a and SCC 25b cell lines revealed either deletions or frameshifts involving TSC2 exon 40 (Table II). To determine if TSC2 down regulation could account for elevated HIF-1α levels during normoxia TSC2 siRNA and a scramble variant were introduced into SCC9 cells that possess wild-type TSC1/TSC2 and manifest low to negligible levels of HIF-1α during normoxia. SCC-4a cells containing a TSC1 exon 17 deletion and a TSC2 exon 40 deletion were also treated in a like manner. These studies resulted in elevated levels of HIF-1α in SCC-9 cells resembling levels observed in SCC-4a and SCC-25b cells, while like treatment of SCC-4a cells produced little change in HIF-1α levels. Noteworthy, was that treatment with the scrambled siRNA did not amass HIF-1α in SCC-9 cells and had no effect in SCC-4a cells. Together, these results indicate that TSC2 loss or mutation may be sufficient for HIF-1α regulation under atmospheric conditions (Figure 6). Transfection of the mutant P582S into SCC-4a cells produced substantially higher levels of HIF-1α than transfections with wild-type HIF-1α. Moreover, the levels of P582S HIF-1α were greater in SCC-4a cells compared to SCC-9 cells, which showed higher levels of HIF-1α than SCC-9 cells transfected with wild-type HIF-1α (Figure 7).

**Table 2:** Summary of SCC cell lines

Cell Lines	TSC1/TSC2	Nucleotide Change	Type of Mutation	HIF-1α Nucleotide Change	Type of Mutation
SSC4a	Exon 17(TSC1) 40 (TSC2) 41 (TSC2)	2111_2112delAT (TSC1); 5238_5255del G5346C	Frameshift in-frame deletion Polymorphism	NC	NC
SSC9	NC	NA	NA	NC	NC
SSC15	NC	NA	NA	NC	NC
SCC25b	Exon 40(TSC2)	5238_5255del	in-frame deletion	NC	NC
UMBSSC11	NC	NA	NA	NC	NC

WD-Well differentiated;MD-Moderately Well-differentiated; UD-Undifferentiated;NC-No change wild-type; P-polymorphism; M-Mutation; del-deletion

**Figure 8:**
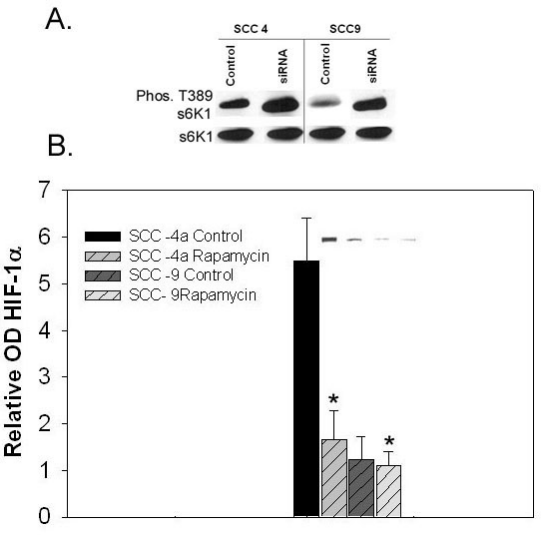
a, b: **The relative levels of HIF-1α and phosphorylation of s6K1 following treatment with Rapamycin in SCC-4a and SCC9 cells. a**)The level of phosphorylation of s6K1 were determined following treatment of SCC-4a and SCC-9 cells with 25 μM Rapamycin for 30 minutes followed by EGF for 1h and then lysed. The cell extracts were subjected to SDS-PAGE and immunoblotting. **b**) Cells were pretreated with 25 μM of Rapamycin as indicated for 30 min, and then exposed to EGF, after 1 hour the cells were harvested and the cell extracts subjected to SDS-PAGE and immunoblotting. Each bar indicates the mean and SD of three repeat wells. Representative gel bands are depicted above the bar graph. (*, P < 0.05)

Recognizing that mTOR, which is down stream of AKT, mediates cell growth and protein synthesis, we employed Rapamycin, a known inhibitor of mTOR, to determine if this signaling pathway affected HIF-1α protein expression. Treatment of SCC 4a and SCC 25b cells under conditions that have been shown to block S6 phorphorylation reduced the levels of phosphorylated S6 (Figure 8) and of HIF-1α to those observed in cell lines with wild type TSC1/TSC2. Interestingly, Rapamycin had little effect in SCC-9 and -15 (not shown) cells both of which possess wild type TSC1/TSC2 (Figure 8).

## Discussion

This study identified 6 cases of tongue SCCs from among 28 cases that manifest HIF-1α heteroduplexes within exon 12 of HIF-1α. Five of the cases were polymorphisms while one case was shown to be a mutation. The two polymorphic variants, P582S and A588T, and the single case presenting as a mutation were the same as those previous described by Tamimoto et al. in a survey of 55 cases of head and neck carcinomas [26]. Interestingly, the same variants have also been described in renal carcinomas, colorectal carcinoma and more recently among prostate cancers [19,38,39]. The cases studied here showed significantly higher levels of HIF-1α by immunohistochemistry, however, there was no correlation between HIF-1α levels with tumor stage or grade according to TNM system. Although previous studies have suggested that the polymorphic variants were always associated with tumors that were greater than T2, the tumors in this study were not part of a progressive series and tumor grading at a single time point was not felt to provide any useful information, albeit all HIF-1α variants were identified in tumors that were graded as T2 or greater [26].

In that the substituted amino acids in the HIF-1α variants identified here are located within or near the N-TAD, interacting with E3 ubiquitin ligase pvHL, it has been suggested that the protein expression levels observed in these tumors and their observed greater transactivation capacity may be due to alteration of the HIF-1α stability. Although previous investigations using COS7 cells found no differences in protein degradation between wild-type HIF-1α and the variant forms [26], more recently it has been demonstrated that P582S HIF-1α stability in CV1 cells was less responsive to iron chelators, which normally promotes HIF-1α accumulation, than were cells possessing wild type HIF-1α. Moreover, these investigators showed that wild-type containing cells rapidly degraded HIF-1 in normoxia while HIF-1α variants maintained protein levels comparable to those observed during hypoxia [19]. Interestingly, proline 582 is not considered as a site for HIF-1α hydroxylation and this site is not been known to promote vHL binding. In fact, site directed substitution of serine for proline at this position has been shown to have no effect on vHL binding after hydroxylation at proline 564 [40]. Although, some have considered that hydroxylation of proline 582 may influence similar post-translational modifications of the full-length HIF-1α molecule or successive steps in the dependent degradation pathway, a more methodological approach will be necessary to settle this point [19].

Recently, we have shown that persistent activation of the Akt pathway was a common event among head and neck squamous carcinomas [41]. Consequently, we explored downstream of Akt to ascertain whether polymorphisms or mutations might explain the elevated levels of HIF-1α. In so doing, nine cases from among the 28 tongue cancers were identified that manifest TSC1 or TSC2 herteoduplexes upon DHPLC analysis. Among these, 8 of the cases contained a polymorphism in exon 41 of the TSC2 gene. This polymorphism, G5346C, has been previously shown to be silent and have no effect on the gene product [42]. One of the cases, patient 18, also had frame-shift deletion in exon 36 and exon 40 of TSC2 as well as being associated with an HIF-1α variant. This case showed extremely high levels of HIF-1α expression suggesting that the two events might complement each other. Interestingly, of the three cells lines that possessed stable high levels of HIF-1α during normoxia, two were shown to contain TSC2 deletion mutations in exon 40 and one of them also contained a frame-shift deletion in exon 17 in TSC1. In that TSC1/TSC2 inhibit mTOR downstream from Akt signaling we sought to substantiate that HIF-1α protein expression was dependent on integrity of the TSC complex. The use of siRNA strategies revealed that targeted degradation of the TSC2 enhanced the levels of HIF-1α in cells containing wild-type TSC2 whereas, the mutant cell line was refractive to such targeted degradation. These findings are in consort with an earlier demonstration that TSC2 regulates VEGF, a HIF-1 target through an mTOR pathway [43].

In solid head and neck squamous carcinomas Rapamycin exhibits a potent anti-tumor effect *in vivo*, inhibiting DNA-synthesis and inducing the apoptotic death of HNSCC cells, which ultimately results in tumor regression. Similar results are not observed for cells grown *in vitro *where the drug appears only to be cytostatic and only reduced cell cycle progression, particularly by delaying their entry into S phase, and by arresting cell lines in G_1 _phase at high doses. Those studies suggested that mTOR may occupy a position at the crossroad of a network of molecular pathways sensing the energy supply, growth promoting or inhibitory factors, the metabolic status of the cells, and the availability of nutrients, and integrates this complex array of incoming information to regulate the synthesis of proteins that are required for cell growth [44]. The studies here showed that treatment with Rapamycin had no effect on HIF-1α levels either during serum-rich or serum deprived condition in cells possessing wild-type TSC1/TSC2, however, in the two cell lines possessing a deletion mutation in TSC2 Rapamycin diminished the levels of HIF-1α. Thus, implying that mutations in the TSC1/TSC2 complex could *in vitro *tip this scale towards an mTOR regulation of HIF-1 mediated cell growth.

There is strong evidence that up regulation of HIF by mTOR is a frequent mechanism regulating tumor growth. Although the oncogenic events that have been associated with mTOR upregulation of HIF have included persistent activation of Akt, overexpression of HER2 and the BCR-ABL, and inactivation of PTEN, similar effects may also be achieved by dysregulation of the TSC1/TSC2 complex by mutation, promoter hypermethylation [45], or by binding with HPV16 E6, which results in the proteasome-mediated degradation of TSC2 [46]. The demonstration here using established cell lines transfected with HIF-1α and the P582S mutant indicates that alterations in HIF-1α and TSC1/TSC2 compliment each other expressing elevated levels of HIF-1α in the face of normal vHL and normoxia. Whether such alterations in the TSC1/TSC2 complex coupled with HIF-1α polymorphisms influence the progression to SCC of the head and neck needs to be further assessed in a larger number of cases and may provide biomarkers to predict responses to specific therapies and overall disease prognosis.

## Materials and methods

### Archival tissue material

Twenty eight primary squamous cell carcinomas (16 males and 12 females) of the tongue tumor were retrieved from the Tumor Bank of the Greenebaum Cancer Center, University of Maryland Baltimore. In that the identity of the subjects was not identifiable by the parameters provided the investigation was judged as exempt by Institutional Review Board.

### Cell lines

Established cell lines of human oral squamous carcinoma cells (SCCs) SCC-4a, -9, -15 and -25 b were obtained or developed (SCC-4a reestablished from SCC-4 xenograph and SCC-25b reestablished from SCC-25 xenograph) from American Type Culture Collection (ATCC) (Manassas, VA) UMBSCC11 was developed at the University of Maryland Baltimore. For these studies, cells were cultured in a 1:1 mixture of Ham's F12 and DMEM containing 10% FBS, 100 U of penicillin, 100 μg/ml streptomycin and 0.4 μg/ml hydrocortisone (Sigma Chemical Co., St. Louis, MO) at 37°C in a 5% CO_2 _air atmosphere (normoxia). Cells were subcultured by disaggregation with trypsin (0.1%)-EDTA (0.01%) in PBS at pH 7.5.

### Protein lysate preparation and western blotting

Cells were plated in 6-well plates using a density of 5 × 10^4 ^cells/well and were allowed to grow to 80% confluence. The cells were washed twice with cold PBS, lysed in RIPA buffer (50 mM Tris [pH 7.4], 150 mM NaCl, 1% Triton X-100, 1% deoxycholic acid, sodium salt, 0.1% sodium dodecyl sulfate [SDS], 100 μg/ml phenylmethylsulfonyl fluoride, 1 μg/ml aprotinin, 1 mM dithiothreitol and 1 mM sodium orthovanadate) for 10 min and scraped. The extracts were centrifuged at 40,000 *g *for 15 min at 4°C. Protein concentrations were measured and equalized using Bio-Rad protein assay (Bio-Rad Laboratories, Richmond, CA) according to the manufacturer's instructions.

Equivalent amounts of protein (50 μg) were then separated by SDS-PAGE and then transferred to polyvinylidene difluoride membranes. Equivalent loading was confirmed by staining membranes with Ponceau-S. The membranes were blocked for 1 h in blocking buffer (1× Tris-buffered saline, 5% nonfat dry milk, and 0.1% Tween 20), which was subsequently replaced by the primary antibody in blocking buffer, overnight at 4°C. After incubation, the membranes were washed three times in washing buffer (1× Tris-buffered saline and 0.1% Tween 20). Primary antibody was detected using horseradish peroxidase-linked goat antimouse (Santa Cruz Biotechnology, Santa Cruz, CA) or goat antirabbit IgG antibody (Santa Cruz Biotechnology) and visualized with SuperSignal West Pico chemiluminescent substrate (Pierce, Rockford, IL). The bands were scanned and quantified using NIH image software.

### Plasmids, HIF-1α protein levels

The P582S HIF-1α mutant was produced by *in vitro *site directed mutagenesis from HA-tagged wild-type HIF-1α in pcDNA3 using Stratagene QuickChange Site-Directed mutagenesis kit, as previously described [19]. To demonstrate the levels of HIF-1α and mutant protein SCC-4a and SCC-9 cells were transfected using the appropriate plasmid with Lipofectamine 2000 (Invitrogene) and the levels assessed by Western blot using anti-HA epitope tag antibodies and visualized with SuperSignal West Pico chemiluminescent substrate (Pierce, Rockford, IL) as described above.

### Immunostaining

Tumor tissue immunostaining for HIF-1α (SC10790), (Santa Cruz Biotech., Inc. Santa Cruz, CA, USA) was performed on 5 μM sections made from the above samples. Staining was analyzed with horseradish peroxidase-linked goat antimouse (Santa Cruz Biotechnology, Santa Cruz, CA) or goat antirabbit IgG antibody (Santa Cruz Biotechnology). The samples were graded either as strongly positive (++++, where more than 70% of tumor cells expressed staining), moderately positive (+++, 50% to 70% positive), Positive (++, 10% to 50% positive staining) or weakly positive (+ < 10% positive staining) Negative and positive controls were included in each batch.

### Real-time RT-PCR assays

Total RNA was extracted from cells by using the RNAqueous kit #1912 (Ambion, Austin, TX, USA) and were treated with DNase. Five μg total RNA was used for first-strand synthesis. cDNA was used for PCR analysis of HIF-1α (forward 5'-CCACAGGACAGTACAGGATG-3' and reverse 5'-TCAAGTCGTGCTGAATAATACC-3'), OS-9 (forward 5'-GGATGATGAAACAGCCAAGG-3' and reverse 5'-GCACATAAGAGCAGGACAAG-3') and 18S rRNA (the 340 base pair fragment of 18s rRNA was used as the housekeeping gene using 5'-AATTGACGGAAGGGCACCAC-3' and 5'-CGGACATCTAAGGGCATCACAG-3' as forward and reverse primers, respectively) [11]. Real-time PCR was performed by using SYBR Green PCR Master Mix and the Applied Biosystems 7000 Real-time PCR Detection System (ABI, Foster, Ca). Expression of HIF-1α mRNA and OS-9 RNA relative to 18S rRNA was calculated based on the threshold cycle (C_T_) for amplification as 2^(ΔCT)^. Melting-curve data for all the samples were obtained to ensure specific amplification.

### Statistical analysis

Data are presented as mean ± SEM. Differences between experiments were analyzed for statistical significance (p < 0.05) by ANOVA or two-sample t tests as appropriate.

### DNA extraction and polymerase chain reaction

Genomic DNA was isolated from peripheral blood mononuclear cells and patient tumor samples to assess polymorphisms versus mutations using GenomicPrep. Cell and Tissue DNA Isolation Kit (Amersham Biosci.; Piscaraway, NJ). as described previously [47]. Genomic polymerase chain reaction (PCR) was carried out in 50- μl reaction volumes containing 100 ng genomic DNA, 0.5 mM primers, 0.2 mM dNTP, 5 ml reaction buffer (100 mM TRIS pH8.3, 500 mM KCl, 15 mM MgCl2, 0.01% gelatin) and 1 U TaqPro Complete DNA polymerase (Danville Scientific; Metuchen, NJ).

To assess HIF-1α we focused on the polymporphisms found in human HIF-1α that resulted in an amino acid substitution within exon 12. Consequently, PCR was performed to amplify the 178-bp fragment of human HIF-1α gene (CATGTATTTGCTGTTTTAAAGGACACAGATTTAGACTTGGAGATGTTAGCTCCCTATATCCCAATGGATGATGACTTCCAGTTACGTTCCTTCGATCAGTTGTCACCATTAGAAAGCAGTTCCGCAAGCCCTGAAAGCGCAAGTCCTCAAAGCACAGTTACAGTATTCCAGCAGACTC) using a primer set, HIF-1α (forward 5'-CAT GTA TTT GCT GTT TTA AAG-3') and HIF-1α (reverse 5'-GAG TCT GCT GGA ATA CTG TAA CTG-3') under the following conditions: 30 cycles of denaturing at 95°C for 30 s, annealing at 61°C for 30 s and extension at 72°C for 30 s. [19,26].

PCR primers for the amplification of *TSC1 *and *TSC2 *exons were as previously described [47]. 28 patient tumor samples and negative (no DNA) control for each amplimer were amplified. Cycling parameters were 95°C for 12 min followed by 33 cycles at 53°C (*TSC2 *exons 2, 4, 5, 8, 11, 14, 15, 16, 17, 19 and 20), 55°C (*TSC1 *exons 3–14;*TSC1 *exons 15–23;*TSC2 *exons 3, 6, 7, 13, 21, 22, 25, 27, 28, 29, 30 and 33 fragment b *TSC2 *exons 1, 9, 10, 12, 23, 24, 31, 33 fragment c, 37, 39, 40 and 41) or 57°C (*TSC2 *exons 18, 26, 32, 33 fragment a, 34, 35, 36 and 38) for 1 min, 72°C for 1 min and 94°C for 1 min, with a final step of 72°C for 10 min.

To assess vHL primer sets for all 3 exons were employed. The primer sequences were as follows: for exon 1 (forward 5'-CGCGAAGACTACGGAGGTCG-3') (reverse 5'GGATGTGTCCTGCCTCAAGGG-3'); exon 2 (forward 5'-ACCGGTGTGGCTCTTAACA-3'0 (reverse 5'-CTTACCACAACAACCTTATCTT-3') and exon 3 (forward 5'-GCCTCTTGTTCGTTCCTTGTACT-3') (reverse 5'-GATCAAGACTCATCAGTACCATC-3').

### Denaturing HPLC

DHPLC was performed utilizing a WAVE DNA fragment analysis system (Transgenomic, Omaha, NE). For these studies five microliters of heteroduplexed PCR fragments was injected onto the DNASep cartridge. Products were eluted at a constant flow rate of 0.9 ml/min with a linear acetonitrile gradient determined by WAVEMaker software (Transgenomic, Omaha, NE) based on the size and GC content of the amplicon. The gradient was achieved by combining 0.1 M triethylammonium acetate (TEAA) buffer (pH 7) (Transgenomic) and Buffer B (0.1 M TEAA with 25% acetonitrile) (Transgenomic). Eluted DNA fragments were detected by the system's UV detector and analyzed as chromatograms. Homo- and heteroduplex peaks were detected between the initial injection peak, produced by residual nucleotides and primers in the reaction, and the wash peak, produced by the acetonitrile flush at the end of each analysis. Melt profiles were constructed using the WAVEMaker software (Transgenomic). A range of partial denaturation temperatures was predicted by this software. PCR products were initially analyzed under nondenaturing conditions, 50°C, to assess the quality of the peak representing both hetero- and homoduplex DNA fragments in a mutant sample. All temperatures within the predicted range for partial denaturation were then assessed for their ability to resolve homo- and heteroduplexes. A single temperature was chosen where all mutants could be resolved.

For HIF-1α DHPLC analyses heteroduplexes were detected at 58.4°C. For *TSC1 *and *TSC2 *a list of the PCR DHPLC run temperatures, run times and ACN gradients used were those indicated by Jones *et al*. [47]. VHL heteroduplexes were detected at 61.5°C.

### DNA sequencing

PCR products displaying an abnormal elution profile or variant DHPLC melt profiles were directly sequenced by using the Sequenase PCR Product Sequencing Kit according to the manufacturer's instructions (Amersham).

## Authors' contributions

CH- Performed transfection and gene expression data.

KN-Performed Western blotting and Real-time PCR

PP- Extracted DNA, and performed DHPLC analyses

RAO-Contributed tissue samples and analyzed clinical data

NGN- Performed gene sequencing and DHPLC studies

JJS- Provided over-all study design, designed probes, transfection vectors, and gene mutations.

## References

[B1] Semenza GL, Wang GL (1992). A nuclear factor induced by hypoxia via de novo protein synthesis binds to the human erythropoietin gene enhancer at a site required for transcriptional activation. Mol Cell Biol.

[B2] Wang GL, Jiang BH, Rue EA, Semenza GL (1995). Hypoxia-inducible factor 1 is a basic-helix-loop-helix-PAS heterodimer regulated by cellular O2 tension. Proc Natl Acad Sci U S A.

[B3] Bruick RK, McKnight SL (2001). A conserved family of prolyl-4-hydroxylases that modify HIF. Science.

[B4] Masson N, Willam C, Maxwell PH, Pugh CW, Ratcliffe PJ (2001). Independent function of two destruction domains in hypoxia-inducible factor-alpha chains activated by prolyl hydroxylation. Embo J.

[B5] Taylor MS (2001). Characterization and comparative analysis of the EGLN gene family. Gene.

[B6] McNeill LA HKSGJMHLEONJMPHPCWRPJSCJ (2002). The use of dioxygen by HIF prolyl hydroxylase (PGD1). Bioorg Med Chem Lett.

[B7] Huang LE, Gu J, Schau M, Bunn HF (1998). Regulation of hypoxia-inducible factor 1alpha is mediated by an O2-dependent degradation domain via the ubiquitin-proteasome pathway. Proc Natl Acad Sci U S A.

[B8] Salceda S, Caro J (1997). Hypoxia-inducible factor 1alpha (HIF-1alpha) protein is rapidly degraded by the ubiquitin-proteasome system under normoxic conditions. Its stabilization by hypoxia depends on redox-induced changes. J Biol Chem.

[B9] Kallio PJ, Wilson WJ, O'Brien S, Makino Y, Poellinger L (1999). Regulation of the hypoxia-inducible transcription factor 1alpha by the ubiquitin-proteasome pathway. J Biol Chem.

[B10] Sutter CH, Laughner E, Semenza GL (2000). Hypoxia-inducible factor 1alpha protein expression is controlled by oxygen-regulated ubiquitination that is disrupted by deletions and missense mutations. Proc Natl Acad Sci U S A.

[B11] Baek JH, Mahon PC, Oh J, Kelly B, Krishnamachary B, Pearson M, Chan DA, Giaccia AJ, Semenza GL (2005). OS-9 interacts with hypoxia-inducible factor 1alpha and prolyl hydroxylases to promote oxygen-dependent degradation of HIF-1alpha. Mol Cell.

[B12] Mahon PC, Hirota K, Semenza GL (2001). FIH-1: a novel protein that interacts with HIF-1alpha and VHL to mediate repression of HIF-1 transcriptional activity. Genes Dev.

[B13] Lando D, Peet DJ, Gorman JJ, Whelan DA, Whitelaw ML, Bruick RK (2002). FIH-1 is an asparaginyl hydroxylase enzyme that regulates the transcriptional activity of hypoxia-inducible factor. Genes Dev.

[B14] Hewitson KS, McNeill LA, Riordan MV, Tian YM, Bullock AN, Welford RW, Elkins JM, Oldham NJ, Bhattacharya S, Gleadle JM, Ratcliffe PJ, Pugh CW, Schofield CJ (2002). Hypoxia-inducible factor (HIF) asparagine hydroxylase is identical to factor inhibiting HIF (FIH) and is related to the cupin structural family. J Biol Chem.

[B15] Dames SA, Martinez-Yamout M, De Guzman RN, Dyson HJ, Wright PE (2002). Structural basis for Hif-1 alpha /CBP recognition in the cellular hypoxic response. Proc Natl Acad Sci U S A.

[B16] Elkins JM, Hewitson KS, McNeill LA, Seibel JF, Schlemminger I, Pugh CW, Ratcliffe PJ, Schofield CJ (2003). Structure of factor-inhibiting hypoxia-inducible factor (HIF) reveals mechanism of oxidative modification of HIF-1 alpha. J Biol Chem.

[B17] Freedman SJ, Sun ZY, Poy F, Kung AL, Livingston DM, Wagner G, Eck MJ (2002). Structural basis for recruitment of CBP/p300 by hypoxia-inducible factor-1 alpha. Proc Natl Acad Sci U S A.

[B18] Li Z, Wang D, Na X, Schoen SR, Messing EM, Wu G (2003). The VHL protein recruits a novel KRAB-A domain protein to repress HIF-1alpha transcriptional activity. Embo J.

[B19] Fu XS, Choi E, Bubley GJ, Balk SP (2005). Identification of hypoxia-inducible factor-1alpha (HIF-1alpha) polymorphism as a mutation in prostate cancer that prevents normoxia-induced degradation. Prostate.

[B20] Hagg M, Wennstrom S (2005). Activation of hypoxia-induced transcription in normoxia. Exp Cell Res.

[B21] Cohen NA, Lai SY, Ziober AF, Ziober BL (2004). Dysregulation of hypoxia inducible factor-1alpha in head and neck squamous cell carcinoma cell lines correlates with invasive potential. Laryngoscope.

[B22] Bos R, Zhong H, Hanrahan CF, Mommers EC, Semenza GL, Pinedo HM, Abeloff MD, Simons JW, van Diest PJ, van der Wall E (2001). Levels of hypoxia-inducible factor-1 alpha during breast carcinogenesis. J Natl Cancer Inst.

[B23] Zhong H, De Marzo AM, Laughner E, Lim M, Hilton DA, Zagzag D, Buechler P, Isaacs WB, Semenza GL, Simons JW (1999). Overexpression of hypoxia-inducible factor 1alpha in common human cancers and their metastases. Cancer Res.

[B24] Koukourakis MI, Giatromanolaki A, Skarlatos J, Corti L, Blandamura S, Piazza M, Gatter KC, Harris AL (2001). Hypoxia inducible factor (HIF-1a and HIF-2a) expression in early esophageal cancer and response to photodynamic therapy and radiotherapy. Cancer Res.

[B25] Kondo K, Kaelin WGJ (2001). The von Hippel-Lindau tumor suppressor gene. Exp Cell Res.

[B26] Tanimoto K, Yoshiga K, Eguchi H, Kaneyasu M, Ukon K, Kumazaki T, Oue N, Yasui W, Imai K, Nakachi K, Poellinger L, Nishiyama M (2003). Hypoxia-inducible factor-1alpha polymorphisms associated with enhanced transactivation capacity, implying clinical significance. Carcinogenesis.

[B27] Law BK (2005). Rapamycin: An anti-cancer immunosuppressant?. Crit Rev Oncol Hematol.

[B28] Rendtorff ND, Bjerregaard B, Frodin M, Kjaergaard S, Hove H, Skovby F, Brondum-Nielsen K, Schwartz M (2005). Analysis of 65 tuberous sclerosis complex (TSC) patients by TSC2 DGGE, TSC1/TSC2 MLPA, and TSC1 long-range PCR sequencing, and report of 28 novel mutations. Hum Mutat.

[B29] Hay N (2005). The Akt-mTOR tango and its relevance to cancer. Cancer Cell.

[B30] Nobukini T, Thomas G (2004). The mTOR/S6K signalling pathway: the role of the TSC1/2 tumour suppressor complex and the proto-oncogene Rheb. Novartis Found Symp.

[B31] Goncharova E, Goncharov D, Noonan D, Krymskaya VP (2004). TSC2 modulates actin cytoskeleton and focal adhesion through TSC1-binding domain and the Rac1 GTPase. J Cell Biol.

[B32] Astrinidis A, Cash TP, Hunter DS, Walker CL, Chernoff J, Henske EP (2002). Tuberin, the tuberous sclerosis complex 2 tumor suppressor gene product, regulates Rho activation, cell adhesion and migration. Oncogene.

[B33] Li S, Braverman R, Li H, Vass WC, Lowy DR, DeClue JE (2003). Regulation of cell morphology and adhesion by the tuberous sclerosis complex (TSC1/2) gene products in human kidney epithelial cells through increased E-cadherin/beta-catenin activity. Mol Carcinog.

[B34] Mak BC, Yeung RS (2004). The tuberous sclerosis complex genes in tumor development. Cancer Invest.

[B35] Henske EP (2004). The genetic basis of kidney cancer: why is tuberous sclerosis complex often overlooked?. Curr Mol Med.

[B36] Knowles MA, Habuchi T, Kennedy W, Cuthbert-Heavens D (2003). Mutation spectrum of the 9q34 tuberous sclerosis gene TSC1 in transitional cell carcinoma of the bladder. Cancer Res.

[B37] Wienecke R, Klemm E, Karparti S, Swanson NA, Green AJ, DeClue JE (2002). Reduction of expression of tuberin, the tuberous-sclerosis-complex-gene-2 product in tuberous sclerosis complex associated connective tissue nevi and sporadic squamous and basal cell carcinomas. J Cutan Pathol.

[B38] Ollerenshaw M, Page T, Hammonds J, Demaine A (2004). Polymorphisms in the hypoxia inducible factor-1alpha gene (HIF1A) are associated with the renal cell carcinoma phenotype. Cancer Genet Cytogenet.

[B39] Kuwai T, Kitadai Y, Tanaka S, Kuroda T, Ochiumi T, Matsumura S, Oue N, Yasui W, Kaneyasu M, Tanimoto K, Nishiyama M, Chayama K (2004). Single nucleotide polymorphism in the hypoxia-inducible factor-1alpha gene in colorectal carcinoma. Oncol Rep.

[B40] Percy MJ, Mooney SM, McMullin MF, Flores A, Lappin TR, Lee FS (2003). A common polymorphism in the oxygen-dependent degradation (ODD) domain of hypoxia inducible factor-1alpha (HIF-1alpha) does not impair Pro-564 hydroxylation. Mol Cancer.

[B41] Amornphimoltham P, Sriuranpong V, Patel V, Benavides F, Conti CJ, Sauk J, Sausville EA, Molinolo AA, Gutkind JS (2004). Persistent activation of the Akt pathway in head and neck squamous cell carcinoma: a potential target for UCN-01. Clin Cancer Res.

[B42] Niida Y, Lawrence-Smith N, Banwell A, Hammer E, Lewis J, Beauchamp RL, Sims K, Ramesh V, Ozelius L (1999). Analysis of both TSC1 and TSC2 for germline mutations in 126 unrelated patients with tuberous sclerosis. Hum Mutat.

[B43] Brugarolas JB, Vazquez F, Reddy A, Sellers WR, Kaelin WGJ (2003). TSC2 regulates VEGF through mTOR-dependent and -independent pathways. Cancer Cell.

[B44] Amornphimoltham PPVSANNGSJJSEAMAAGJS mTOR, a Molecular Target in Squamous Cell Carcinomas of the Head and Neck. Clin Cancer Res.

[B45] Jiang WG, Sampson J, Martin TA, Lee-Jones L, Watkins G, Douglas-Jones A, Mokbel K, Mansel RE (2005). Tuberin and hamartin are aberrantly expressed and linked to clinical outcome in human breast cancer: the role of promoter methylation of TSC genes. Eur J Cancer.

[B46] Lu Z, Hu X, Li Y, Zheng L, Zhou Y, Jiang H, Ning T, Basang Z, Zhang C, Ke Y (2004). Human papillomavirus 16 E6 oncoprotein interferences with insulin signaling pathway by binding to tuberin. J Biol Chem.

[B47] Jones AC, Sampson JR, Hoogendoorn B, Cohen D, Cheadle JP (2000). Application and evaluation of denaturing HPLC for molecular genetic analysis in tuberous sclerosis. Hum Genet.

